# Association of T and NK Cell Phenotype With the Diagnosis of Myalgic Encephalomyelitis/Chronic Fatigue Syndrome (ME/CFS)

**DOI:** 10.3389/fimmu.2018.01028

**Published:** 2018-05-09

**Authors:** Jose Luis Rivas, Teresa Palencia, Guerau Fernández, Milagros García

**Affiliations:** ^1^ME/CFS Unit, ASSSEMBiomédics, Barcelona, Spain; ^2^Bioinformatics Unit, Genetics and Molecular Medicine Service, Hospital Sant Joan de Déu, Barcelona, Spain; ^3^Immunology Department, Biomedical Diagnostic Center, Hospital Clínic Barcelona, Barcelona, Spain

**Keywords:** chronic fatigue syndrome, natural killer cells, T regulatory cells, NKp46, NKG2C, diagnosis, biomarker

## Abstract

Myalgic encephalomyelitis/chronic fatigue syndrome (ME/CFS) is a pathological condition characterized by incapacitating fatigue and a combination of neurologic, immunologic, and endocrine symptoms. At present its diagnosis is based exclusively on clinical criteria. Several studies have described altered immunologic profiles; therefore, we proposed to further examine the more significant differences, particularly T and NK cell subpopulations that could be conditioned by viral infections, to discern their utility in improving the diagnosis and characterization of the patients. The study included 76 patients that fulfilled the revised Canadian Consensus Criteria (CCC 2010) for ME/CFS and 73 healthy controls, matched for age and gender. Immunophenotyping of different T cell and natural killer cell subpopulations in peripheral blood was determined by flow cytometry. ME/CFS patients showed significantly lower values of T regulatory cells (CD4^+^CD25^++(high)^FOXP3^+^) and higher NKT-like cells (CD3^+^CD16^+/−^CD56^+^) than the healthy individuals. Regarding NK phenotypes, NKG2C was significantly lower and NKCD69 and NKCD56 bright were significantly higher in the patients group. A classification model was generated using the more relevant cell phenotype differences (NKG2C and T regulatory cells) that was able to classify the individuals as ME/CFS patients or healthy in a 70% of cases. The observed differences in some of the subpopulations of T and NK cells between patients and healthy controls could define a distinct immunological profile that can help in the diagnostic process of ME/CFS patients, contribute to the recognition of the disease and to the search of more specific treatments. However, more studies are needed to corroborate these findings and to contribute to establish a consensus in diagnosis.

## Introduction

Myalgic encephalomyelitis/chronic fatigue syndrome (ME/CFS) is a pathological condition characterized by incapacitating fatigue for several months not remitting with rest and a combination of symptoms based on neurologic, immunologic, and endocrine disturbances (1, 2), such as post-exertional malaise, pain, unrefreshing sleep, cognitive impairment, orthostatic intolerance, flu-like symptoms, anxiety, and depression, among others. It is defined as a neurological disease (ICD G93.3) by the World Health Organization despite being a multisystemic condition.

There are no specific diagnostic tests consequently many patients with the disorder are not diagnosed or suffer many delays. There are several case definitions, Fukuda Case Definition for CFS (CDC 1994) (1), Canadian Consensus Criteria (CCC) for ME/CFS (CCC 2003) (3), NICE Clinical Guidelines for CFS/ME (2007), and Revised CCC for ME/CFS (CCC 2010), International Consensus Criteria for ME (ICC 2011) (2) each one displaying different diagnostic criteria. More recently, the Institute of Medicine has proposed a more simplified criteria (4). Therefore, it is crucial that the diagnosis can be supported on objective tests.

The etiopathogenic processes of the disease are still largely unknown. Infectious agents such as diverse herpes-virus like Epstein–Barr virus (EBV) (5–8), cytomegalovirus (CMVH) (8), herpes simplex 6 (HHV-6) (8, 9), other viruses like enterovirus (10, 11), parvovirus B19 (12, 13), murine leukemia virus (XMRV) (14–16), and other microorganisms, such as *Borrelia* sp. (17, 18) have been postulated as triggering factors to no avail. Treatment with antiviral drugs at high doses, Valaciclovir for EBV (19, 20) and Valganciclovir for EBV and HHV (21, 22) have shown some improvement.

The potential role of the microorganisms has driven the research into the immune factors and attempts to characterize the immune profile of ME/CFS, with great heterogeneity of results (23).

Altered cytokine profiles have been observed in ME/CFS patients although more indicative of immune activation and inflammation than specific for ME/CFS (24), and a cytokine plasma signature has been observed in the early stages of the disease correlating better with illness duration than with measures of illness severity, suggesting that the immunopathology of ME/CFS is not static (25). Also described has been a significantly lower expression of the CD69 activation marker on T cells and on NK cells in ME/CFS patients than in healthy subjects (26), altered NK subpopulations and functional capacity (26–29), showing a defect in T- and NK cell activation in these patients (23). However, results are discrete in some and in other studies and there are no observed differences (30–32).

Recently, Theorell et al. (33) evaluated cytotoxic lymphocyte phenotype and function in ME/CFS and found no differences in the number of cytotoxic T cell and adaptive NK cell subsets, exocytosis, pro-inflammatory cytokine production, and adrenalin inhibition compared to matched healthy controls.

A previous study by Curriu et al. (34), however, showed differences in the phenotype and proliferative responses of T cells and NK cells that clearly clustered CFS individuals and could be useful to identify these patients. The patients also had increased levels of T regulatory cells (CD4^+^CD25^+^FOXP3^+^) and lower proliferative responses *in vitro* and *in vivo*. CD8^+^ T cells from the CFS group showed significantly lower activation and frequency of effector memory cells.

In this context, due to the relevance that these immunophenotypes could have as an aid in the diagnosis of ME/CFS, we decided to evaluate whether those results could be reproduced in a study with an extended cohort and stringent clinical criteria to overcome one of the limitations of the majority of the immune phenotyping studies, that is the small number of patients and controls. Our main aim was to assess potential lymphoid cell phenotypes associated with ME/CFS pathology that could help in the diagnosis. Other objectives were to study whether there was an association between any of the cell phenotypes and severity of the disease, subgroups of patients or viral serologies (HCMV, EBV).

## Materials and Methods

### Ethics

This study was carried out in accordance with the recommendations of *Ley General de Sanidad (25/4/1986) Art. 10*, with written informed consent from all subjects.

The study was approved by the Healthcare Ethics Committee of the Hospital Clinic de Barcelona HCB/2015/0870. All participants gave written informed consent, complying with current legislation.

### Patient Population

The study included 76 ME/CFS patients and 73 healthy controls, matched for age and gender. All the participants were over 18 years old. The patients were selected among patients with a diagnosis of ME/CFS, members of a group dedicated to the research, diagnosis, and treatment of ME/CFS in Barcelona, Madrid, and San Sebastian (ASSSEMBiomedics), that fulfilled the revised Canadian Consensus Criteria (CCC 2010) assessed by two medical practitioners and following a questionnaire.

Patients who under the assessment of one of the medical practitioners were considered as not meeting the revised CCC 2010 were excluded, as well as patients with a medical condition other than ME/CFS that could justify the symptoms of the disease. Exclusion criteria for the control group was to be a first or second degree relative of a ME/CFS patient.

### Assessment of the Severity of Symptoms

Evaluation of the burden of the disease was by a self-reported Short Form 36 (SF-36) questionnaire (35), an 8-scale profile including physical and mental functional health, and one of the most used generic surveys to evaluate the quality of life related to health. The level of fatigue was assessed by a Scale of Degree of Impairment proposed by J. Fernández Solà (36) that rates fatigue in four levels according to the degree of impact in the quality of life and daily activities of the patient (grade 1 <20%, grade 2 30–50%, grade 3 >50%, and grade 4 bedbound). Furthermore, ME/CFS patients were asked to grade the proportion of fatigue vs pain disrupting their daily activities.

### Immunophenotyping

Peripheral whole blood was collected from all participants and analyzed within 6 h of collection. Collection of samples was in groups of 17–24 individuals, a combination of patients and healthy controls. We were not able to obtain an equal number of patients and controls for every group but tried to get the closest to 50% (patient’s median 53.8%, range 18–85%). Lymphocyte phenotyping was performed in fresh blood on the BD FACSCanto (Becton Dickinson, US) cytometer, with protocols designed for the purpose of the study. Cells were labeled with the following fluorochrome conjugated monoclonal antibodies: CD5-FITC (Beckman Coulter, CA, US), CD8-PE, CD25-PECy5.5, CD127-PECy7, CD3-APCH7, CD4-BV421, CD45-BV510, CD85j (IL-T2)-FITC, CD16-PE, CD56-PE, CD8-PerCP5.5, NKp46-APC, CD45-APC-H7, CD3-BV421, CD57-FITC, CD45-PerCP-Cy5.5, CD56-PECy7, CD69-APC-Cy7 (Becton Dickinson Biosciences, CA, US), FoxP3-APC, CD159c (NKG2C)-PECy7, and CD159a (NKG2A)-APC (MACS Miltenyi Biotec, Germany). Cells were incubated at 4°C protected from light for 30 min. Then, samples were lysed with FacsLysing (Becton Dickinson Biosciences, CA, USA), for 10 min, and washed twice with PBS. A live/death cell marker was not used, but we aimed to reduce this limitation by using gates based on the forward and side scatter to remove debris and dead cells to the maximum possible. We also used pulse geometry gate (FSC-H × FSC-A) to eliminate doublets that could have been originated by dead cells clumps. CD45^+^ was used to determine lymphocyte population. Data were analyzed using the *BD FACSDiva Software*.

A complete full blood count was also conducted to allow for approximation of absolute counts of the phenotyped subpopulations.

### Assessment of CMVH and EBV Antibodies

Venous blood was collected in standard gel separator tubes. IgG antibodies to human cytomegalovirus (HCMV) were measured by chemiluminescence on Architect I2000SR (Abbott Diagnostics, EUA). Quantification of Epstein virus capsid antigen IgG antibodies (EBV VCA) was carried out by enzyme immunoassay [Captia EBV VCA (P-18) IgG, Trinity Biotech, Ireland].

### Statistical Analysis

We used principal component analysis (PCA) to identify the components that maximize variance and determine whether defined patterns correlate with the already defined groups or if batch effect could be a leading cause of variability. Differential cell population between controls and chronic fatigue patients was determined using the non-parametric Mann–Whitney test (GraphPad.Prism.V.5.00), followed by multiple corrections using a false discovery rate threshold of 5% (*p*-adjusted <0.05). Cell population correlation was determined using Spearman’s rho. To determine significant differences between correlations we used the paired *r* function from the psych R package (*p* < 0.01).

To establish meaningful variables in the classification model we used Weka software bundle (3.8.0). To select relevant attributes for the model we used as attribute evaluator CfsSubsetEval and BestFirst as search method. Once the attributes were selected and in order to check the relevance of all attributes, we followed a leave one out strategy and checked for significant reduction of model accuracy. We defined accuracy as (True Positive + True Negative)/(True Positive + True Negative + False Positive + False Negative). Evaluation was performed with 10-fold cross-validation to reduce model overfitting due to the fact that we were averaging 10 different training sets that represented the entire data set. The purpose of the test is to be used as an objective aid in support of the diagnosis in patients with high degree of clinical suspicion for ME/CFS.

## Results

### Demographic Characteristics of Patients

The group of cases included 76 patients and the control group 73 healthy individuals. There were no differences in age or gender between the groups (Table 1). Mean age for the patients was 49.78 vs 48.71 years for the control group, and 82.89% were female vs 82.19% in the control group. Mean duration of symptoms was 17.44 years (5–63). The symptoms began acutely in 76.3% of the patients and infection was the trigger in 41.37% of these patients.

**Table 1:** Main demographic characteristics of the participants.

	Cases *n* = 76	Controls *n* = 73	*p* Value
Age (mean years)	49.78 (20–80)	48.71 (23–66)	ns
Gender (*n*, % women)	63 (82.89)	60 (82.19)	ns
Sudden onset (*n*, %)	58 (76.3)		
Infection as trigger (*n*, %)	24/58 (41.37)		
Mean years of symptoms (from sudden onset)	17.44 (5–63)		
Short form 36 (0–100) (normal >50)	32.83 (6–64)		
Mean degree of fatigue (1–4)	2.28 (1.80–2.70)		
% fatigue vs pain (mean)	76 (60–98.75)		

### T and NK Cell Phenotypes

**Figure 1:**
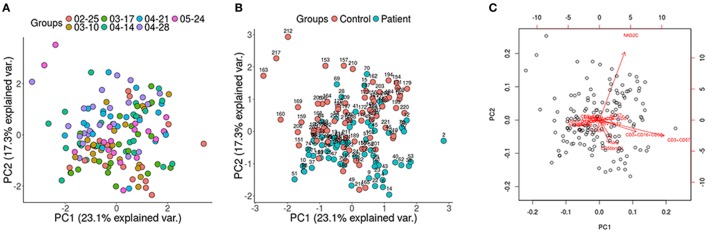
Principal component analysis (PCA) displaying all the participants of the study. **(A)** No differences between groups regarding the extraction date; **(B)** control and patients samples were analyzed using cell population data based on differential markers determined by FACS, showing the patients (blue) toward the lower right end of the plot, whereas the control group (red) is clustered toward the upper left of the plot; **(C)** highlighted are the variables that spread the samples in specific directions within the PCA (red arrows).

Immunophenotyping was performed in batches of 17–24 individuals. PCA of the data by groups was carried out in order to evaluate whether a bias had occurred during the extraction of the samples. No differences were observed between the groups regarding the extraction (Figure 1A). To determine the presence of non-biological variation between groups due to extraction date we performed a guided principal component analysis (gPCA). gPCA generates a delta statistic that quantifies the proportion of variance due to batch effects. We generated 1,000 delta values by permutation of our data. This delta values were plotted and compared with the delta value generated taking into account our extraction grouping (Figure S1 in Supplementary Material). We can observe that our delta value falls within the permutated distribution showing no batch effect due to extraction date.

**Figure 2:**
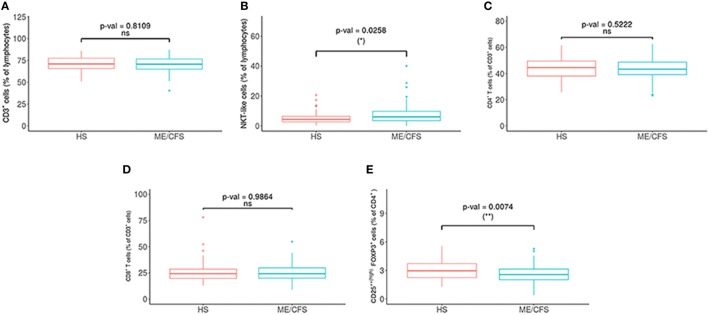
Comparison of lymphocytes subsets in myalgic encephalomyelitis/chronic fatigue syndrome (ME/CFS) patients (*n* = 76) and healthy subjects (*n* = 73). **(A)** Percentages of T (CD3^+^) and **(B)** NKT (CD3^+^CD16^+/−^CD56^+^) cells were analyzed in gated CD45^+^ lymphocytes. **(C,D)** Percentages of CD4^+^ and CD8^+^ were obtained after gating CD3^+^ lymphocytes. **(E)** The percentage of T regulatory cells [CD25^++(high)^FoxP3^+^] was obtained after gating CD4^+^ T cells. Figures show median values (lines), interquartile ranges (boxes), and 10–90 percentile values (bars). *p* Values obtained by Wilcoxon non-parametric test.

We obtained the percentage and absolute numbers of CD4 and CD8 T cells, T regulatory cells (CD4^+^CD25^++(high)^FOXP3^+^), and different NK subpopulations: NKT-like cells (CD3^+^CD16^+/−^CD56^+^), NK cells (CD3^−^CD16^+/−^CD56^+^), NK CD56^++(high)^, NK CD56^+(dim)^, NKCD57^+^, CD69^+^, NKp46^+^, NKG2C^+^, NKG2A^+^, and ILT2^+^ (Figure 2; Figure S2 in Supplementary Material).

**Figure 3:**
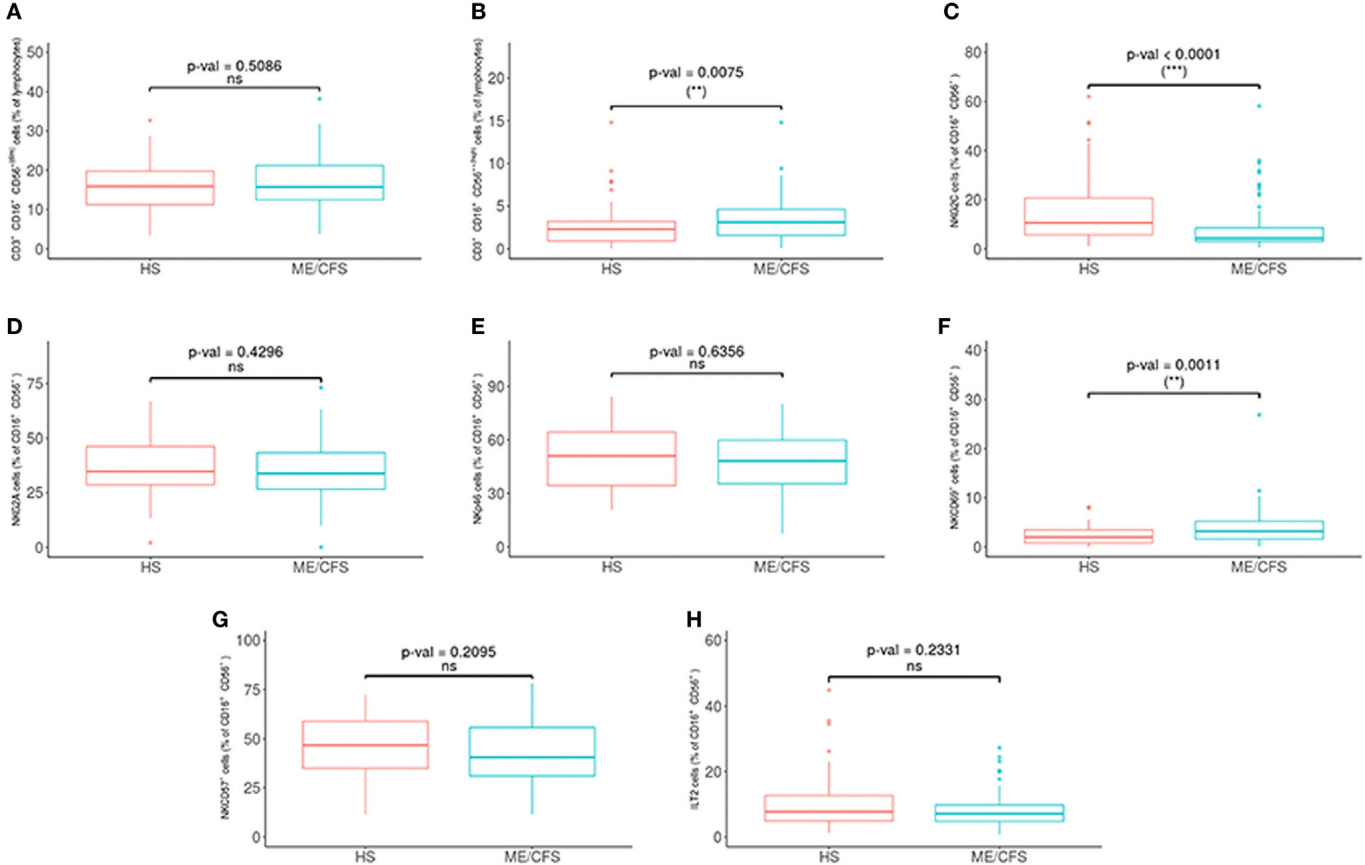
Analysis of NK cell subsets in myalgic encephalomyelitis/chronic fatigue syndrome (ME/CFS) patients (*n* = 76) and healthy subjects (*n* = 73). **(A,B)** NK cells as CD16^+/−^CD56^+(dim)^ and CD16^+/−^CD56^++(high)^ were obtained in gated CD45^+^ lymphocytes. **(C–H)** Percentages of NKG2C, NKG2A, NKp46, NKCD69, NKCD57, and ILT2 NK cells were obtained after gating for CD16^+/−^CD56^+^ lymphocytes. Figures show median values (lines), interquartile ranges (boxes), and 10–90 percentile values (bars). *p* Values obtained by Wilcoxon non-parametric test.

**Figure 4:**
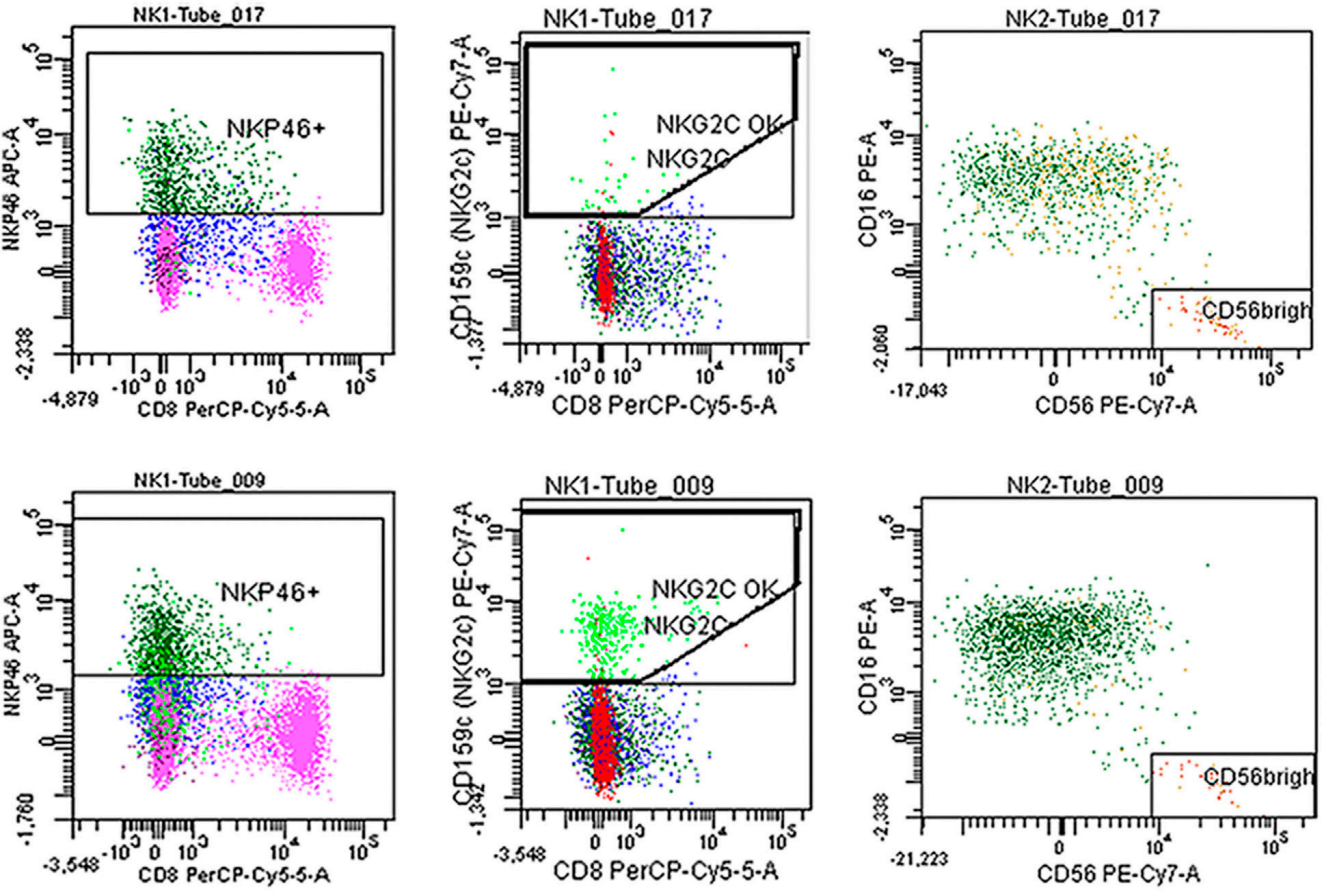
Fresh blood was stained with the antibodies described in Section “Materials and Methods.” Representative plots of NKp46, NKG2C, and CD56^++(high)^ of a myalgic encephalomyelitis/chronic fatigue syndrome patient (tube 017) and a healthy control (tube 009).

Potential differences in the variables between the patients and the healthy controls were analyzed with a PCA (Figures 1B,C) and a Wilcoxon non-parametric test. ME/CFS patients showed significantly lower values of T regulatory cells and higher NKT-like cells than the healthy individuals (*p* = 0.0074 and *p* = 0.0258, respectively) (Figure 2). Regarding NK phenotypes, NKG2C was significantly lower (*p* < 0.0001) and NKCD69 and NKCD56^++(high)^ were significantly higher in the patients group (*p* = 0.0011 and *p* = 0.0075, respectively) (Figures 3 and 4; Figure S3 in Supplementary Material).

### Correlation Between Cell Phenotypes

**Figure 5:**
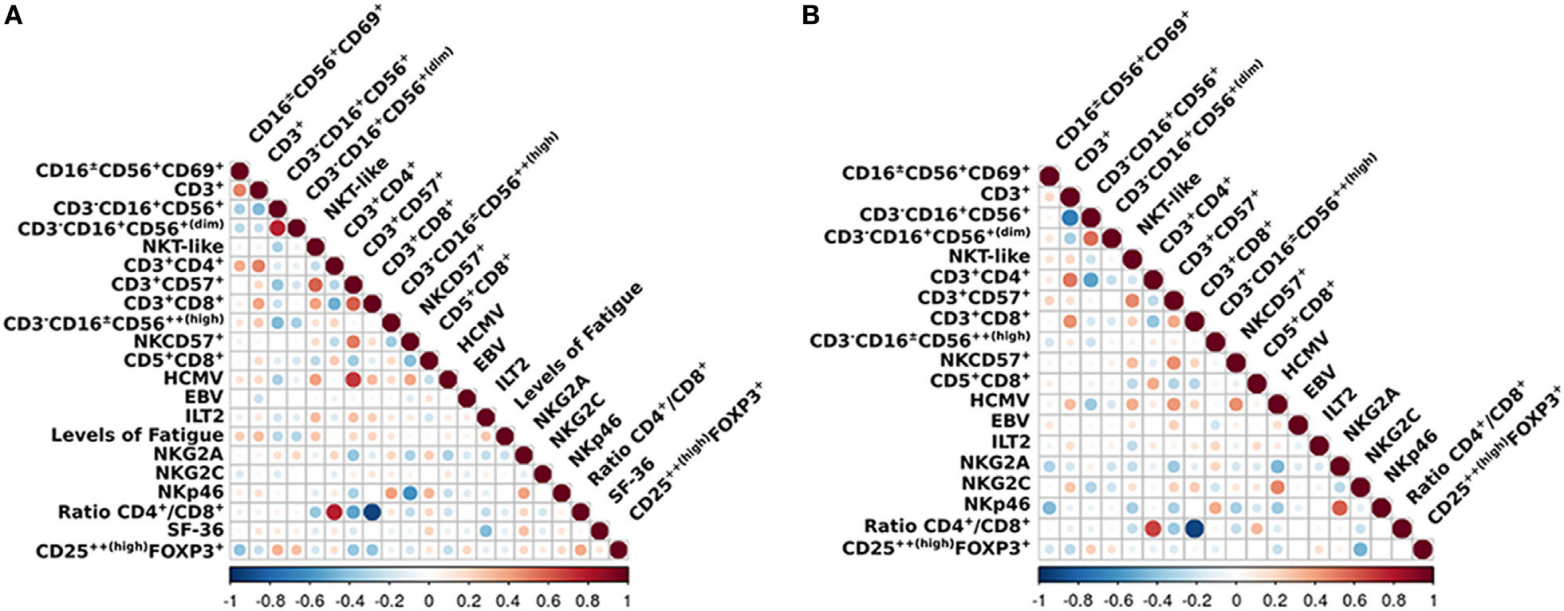
Overview of the correlations of the analized immunophenotypes. Differences between **(A)** myalgic encephalomyelitis/chronic fatigue syndrome patients and **(B)** healthy controls. Color marks positive (red) or negative (blue) correlation. The size of the circle indicates the scale of the correlation.

**Figure 6:**
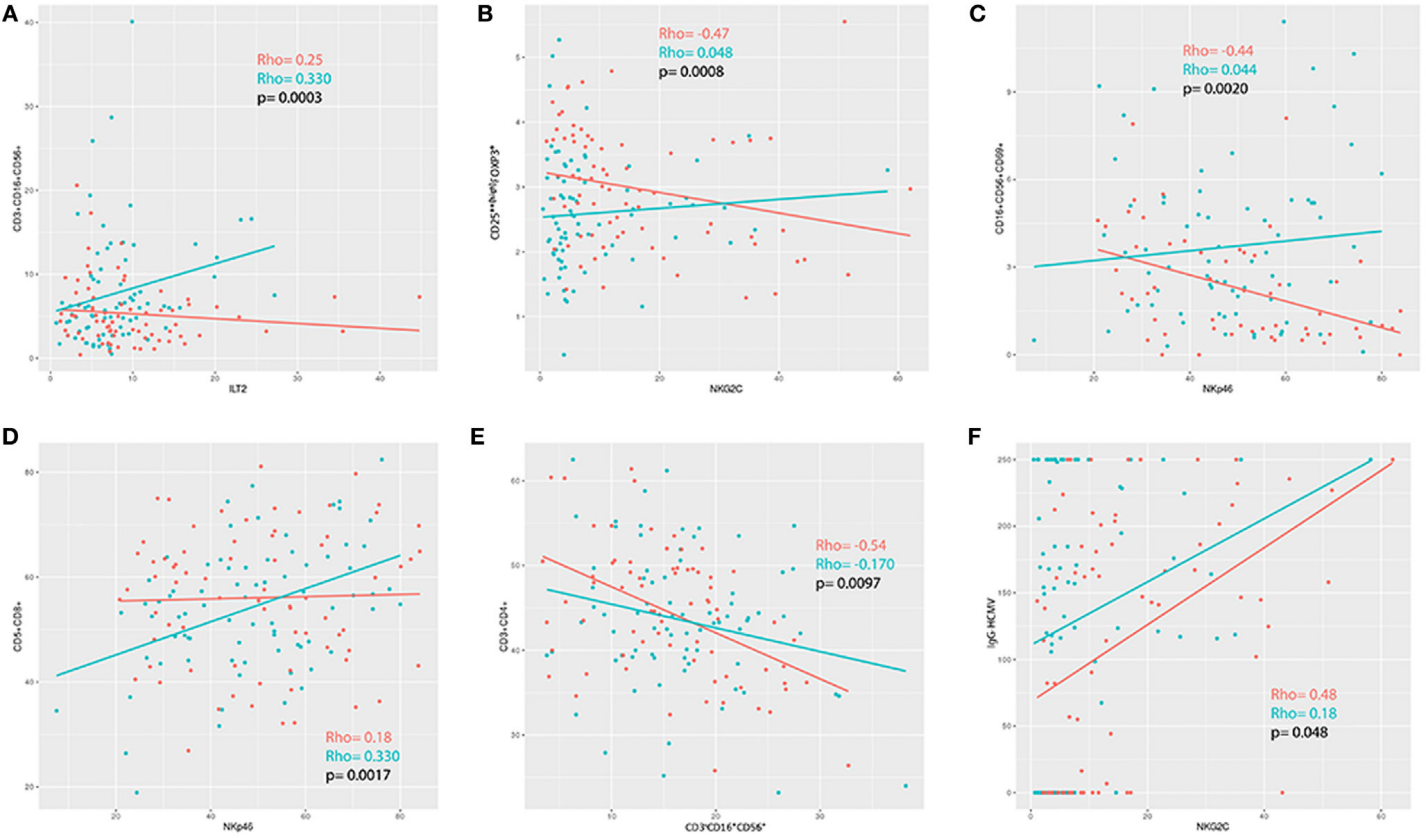
**(A–E)** Correlation analysis between immunophenotypes in patients (blue) and healthy controls (red). **(F)** Positive correlation between NKG2C and IgG-HCMV for both patients and controls, although more pronounced in the patients (controls rho = 0.48; myalgic encephalomyelitis/chronic fatigue syndrome rho = 0.18; *p* = 0.048).

We applied a *z*-transformation to scale data previous to determination of correlation (Spearman rho) between the different cell phenotypes. To determine significant differences between correlations a paired *r* function from the psych R package (*p* < 0.01) was used. We observed that the correlations between ILT2 and CD3^+^CD16^+/−^CD56^+^, T regulatory cells and NKG2C, NKP46 and CD16^+/−^CD56^+^CD69^+^, NKP46 and CD5^+^CD8^+^, and NK cells and CD3^+^CD4^+^ were significantly different in the ME/CFS individuals compared to the healthy subjects (*p* = 0.0003, *p* = 0.0008, *p* = 0.0020, *p* = 0.0017, and *p* = 0.0097, respectively) (Figures 5 and 6).

### ME/CFS Diagnosis Probability Based on Cell Phenotype Differences

To explore the possibility for the cell phenotype, significant differences observed between patients and healthy subjects to be used in ME/CFS diagnosis, we used Weka 3.8.0 software to generate a classification model. To select relevant attributes for the model, we used as attribute evaluator CfsSubsetEval and BestFirst as search method. Evaluation was performed with 10-fold cross-validation. The most informative cell phenotypes generated were NKG2C (10-folds), T regulatory cells (threefold), CD16^+/−^CD56^+^CD69^+^, NKCD56^++(high)^ (twofold), CD3^+^CD57^+^ and CD3^+^CD16^+/−^CD56^+^ (onefold).

**Figure 7:**
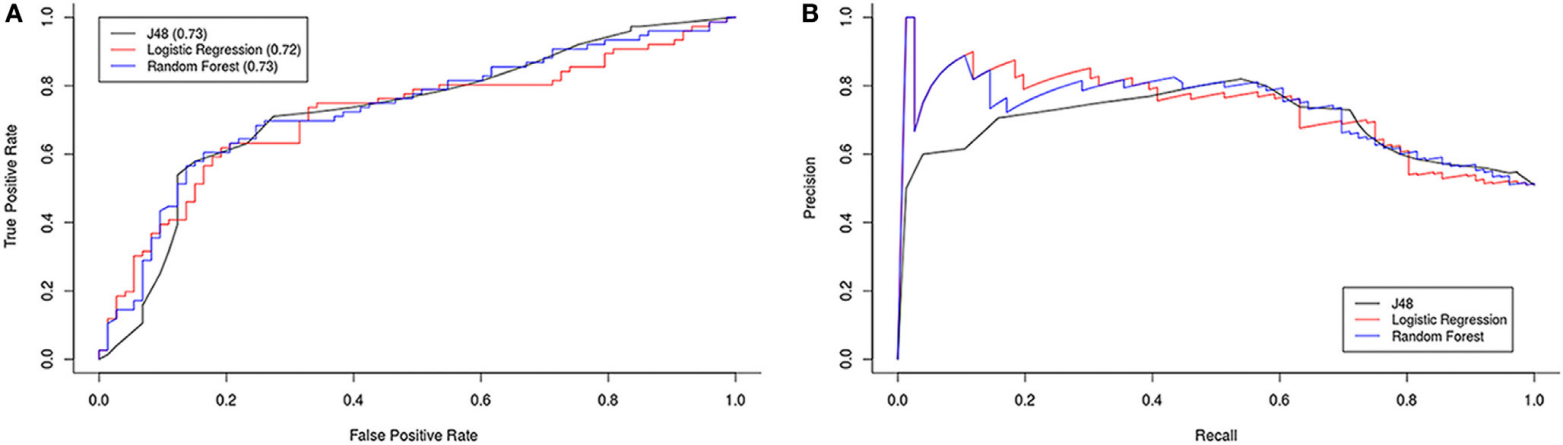
ROC **(A)** and precision/recall **(B)** graphs for all three classification models.

Using these attributes we tested three different models (logistic regression, J48, and Random Forest) with 10-fold cross-validation. The minimum best model used T regulatory cells and NKG2C with approximately 70% accuracy, i.e., we were able to classify the individuals as ME/CFS patients or healthy with those two attributes in a 70% of cases. We obtained ROC and precision/recall graphs for all three classification models (Figure 7).

### SF-36 Questionnaire and Levels of Fatigue

Patients responded to the Short Form 36 Health Questionnaire (SF-36 HQ), one of the most widely used and generic health-related quality of life questionnaires. In our experience, the SF-36 does not portray well the effect of the fatigue in the quality of life in ME/CFS patients. For that reason, we also used a survey to assess the level of fatigue based on the fatigue evaluation questionnaire developed by Fernández-Sola and modified the purpose of the study. This questionnaire rates the fatigue in four levels according to the degree of impact in the quality of life and daily activities of the individual.

Mean value for the SF-36 HQ was 32.83 (6–64), and for the level of fatigue was 2.28 (1.80–2.70) (Table 1). The relationship between both questionnaires showed a moderate correlation (rho = −0.46). The values of SF-36 decreased as the levels of fatigue increased, as expected (Figure S4A in Supplementary Material).

We analyzed the relationship between cell phenotypes and severity of fatigue and SF-36 questionnaire values, but we did not observe a significant correlation between any of the cell phenotypes and the measurements of quality of life and degree of fatigue. However, we observed a positive trend between the level of fatigue and NKT-like cells (CD3^+^CD16^+/−^CD56^+^) (rho = 0.30), and a negative trend between the percentage of fatigue vs pain and NKG2C expression (rho = −0.33) (Figures S4B,C in Supplementary Material).

### Evaluation of Subgroups of ME/CFS Patients and Cell Phenotypes

There were three groups of patients according to the different onset of the symptoms. Some patients referred the initial symptoms soon after an infectious process (*n* = 24); others described the symptoms not related to infections (*n* = 37). A third group (*n* = 15) did not remember how the symptoms started; therefore, we exclude them from this analysis.

We compared the cell phenotypes of these two groups vs the healthy individuals using a Wilcoxon non-parametric test. We observed significant differences between the groups with regards to CD56^++(high)^ (*p* = 0.1559 and *p* = 0.0036 for the “infection” and “no infection” onset groups, respectively) and CD3^+^CD16^+/−^CD56^+^ cells (*p* = 0.0243 and *p* = 0.1041, respectively) (Table 2).

**Table 2:** Comparison of differences in the cell phenotypes found to be significantly different (in bold) in myalgic encephalomyelitis/chronic fatigue syndrome (ME/CFS) patients according to the presence or absence of an infectious process as a trigger for the ME/CFS symptoms.

	Infection (*n* = 24) vs no inf (*n* = 37)	Infection (*n* = 24) vs HD (*n* = 73)	No infection (*n* = 37) vs HD (*n* = 73)	Total ME/CFS (*n* = 76) vs HD (*n* = 73)
NKT	0.4294	**0.024**	**0.1041**	0.0258
CD56^++(high)^	0.2810	**0.1559**	**0.0036**	0.0075
CD69^+^	0.8168	0.0245	0.0037	0.0011
NKG2C	0.3877	0.0039	0.0002	<0.0001
Treg	0.1265	0.0025	0.0249	0.0074

### Evaluation of Correlation Between Viral Serology and Cell Phenotypes

Levels of IgG anti-HCMV and anti-EBV VCA were determined by chemiluminescence (Architect I1000SR/I2000SR, Abbott Diagnostics, EUA) and ELISA [Captia EBV VCA (P-18) IgG, Trinity Biotech] respectively.

There were no differences between the patients and healthy individuals with regards to the prevalence of the positivity of the antibodies (Table 3). We observed a positive correlation between NKG2C and IgG-HCMV for both patients and controls, however, there was a significant difference regarding the strength of this correlation (*p* = 0.048) (Figure 6F).

**Table 3:** Prevalence of positive and negative IgG anti-Epstein–Barr virus (EBV) VCA and CMVH serology, in myalgic encephalomyelitis/chronic fatigue syndrome (ME/CFS) patients and healthy subjects.

		ME/CFS (*n* = 76)	HS (*n* = 73)
EBV	POS	72 (94.7%)	68 (93.15%)
	NEG	4 (5.2%)	5 (6.8%)
CMV	POS	53 (69.7%)	50 (68.4%)
	NEG	23 (30.2%)	23 (31.5%)

## Discussion

Previous studies have evaluated immune profiles in ME/CFS looking for potential biomarkers. Some have found a reduced NK cell cytotoxic activity (27, 28, 37–40) that has not been corroborated in other studies (30–33). Theorell et al. (33) argue that the cytotoxic activity could be reduced when using whole peripheral blood (41, 42) or PBMC immediately obtained (23, 43) but could appear normal when using frozen PBMC. Also other soluble factors like cytokines, catecholamines, and hormones would decrease according to the time lapse, since obtaining the sample that could explain the different results obtained in different studies. We used whole peripheral blood analyzed within 6 h of collection that may have allowed for detection of changes that could be lost when using a more processed sample.

We observed in the ME/CFS patients an increased NK CD56^++(high)^ population (*p* = 0.0075), a small group of NK cells (maximum 10%) with greater cytokine secretion capacity, particularly IFNγ, and with low cytotoxic activity (44, 45). This would be in keeping with the observations by Tireli et al. (46), in a study with 40 patients and 35 healthy controls. Also, Hardcastle et al. (44, 45) observed increased NK CD56^++(high)^ levels in patients with greater severity of the disease compared to the moderately affected patients. However, the same group had observed lower NK CD56^++(high)^ cell numbers in two previous studies with 95 and 10 patients, respectively (23, 37), and in another study the reduced NK CD56^++(high)^ cell numbers were related to the time course of the disease (28). NK CD56^++(high)^ cells are more resistant to apoptosis than NK CD56^+(dim)^ cells (47), so have a longer life span and can induce T cell proliferation that could lead to autoimmunity (48) and contribute to inflammation (49, 50).

Interestingly, we observed increased levels of NKT-like cells and lower NK CD56^++(high)^ cells in the group of patients that had described an infection before the onset of the disease. These populations have a role in the regulation of the immune response through their cytokines. While NK CD56^++(high)^ is relevant to regulate anti-viral and anti-intracellular infections, NKT cells can play a role in Th2 immune responses. However, higher levels of NK CD56^++(high)^ cells in the “no infection” onset group could be due to the exposure to raised levels of catecholamines secondary to the chronic activation of the hypothalamic–pituitary–adrenal axis as described by Loebel et al. (51).

No significant difference was found in our study for the NKp46 expression in percentage and absolute numbers (*p* = 0.6556 and *p* = 0.3897) between patients and healthy individuals, in contrast to the observations by Curriu et al. (34) who observed increased values of NKp46 expression in patients compared to the healthy controls.

NKp46 together with NKp30 and NKp44 are natural cytotoxicity receptors (NCRs) (52) involved in viral and tumoral cell lysis (53) interacting with non-MHC receptors and without the seeming need of MHC class I antigenic stimulation. Increased levels of these receptors have been observed in autoimmune/inflammatory conditions, such as Sjögren (54) and Crohn’s disease (55) while lower levels have been described in infections like HIV (56), tuberculosis(57), human influenza virus (58), VHC (59), and chronic CMVH (60, 61).

NKG2C is a NK lectin-like activating receptor that recognizes the non-classical human leukocyte antigen E (HLA-E). In our study, we observed a lower expression in ME/CFS patients (*p* < 0.0001), in contrast to the findings by Theorell et al. (33), that did not observe differences in NKG2C expression in ME/CFS patients. This could be due to the different methodology used (whole peripheral blood vs frozen PBMC in their study) (62) and their smaller cohort analyzed.

According to Lopez-Botet et al. and Malmberg et al. (60, 61, 63–68) expression of this receptor is increased in chronic infection by HCMV, but also in coinfection with EBV (69, 70) and other virus (71–79). The cell population that characterizes chronic HCMV infection would show increased levels of NKG2C activating receptor, decreased levels of NKp30 and NKp46 receptors, increased levels of inhibitory receptors KIR and CD85j (ILT2/LIR-1), and decreased levels of the inhibitory receptor NKG2A (60, 63–66, 80). The immunophenotype for acute and latent CMVH infection would be NKG2C^+^ NKG2A^+^ ILT2^+^ NKp46^−^ NKp30^−^ NKCD57^+^ (61, 64, 67, 68, 81). Gumà et al. also describe this profile in asymptomatic individuals with positive HCMV serology (64). Lopez Vergés et al. (82) observed that during acute CMV infection, the NKG2C^+^ NK cells proliferated, became NKG2C^hi^, and finally acquired CD57, proposing that CD57 might provide a marker of “memory” NK cells that have been expanded in response to infection.

We did not observe significant differences with regards to the percentage nor absolute numbers of CD57^+^ NK cells between ME/CSF patients and healthy individuals (*p* = 0.2095 and *p* = 0.874, respectively), as shown by other studies (33, 83). However, there are other studies showing lower levels of this cell population. CD57 expression has been considered as a marker of senescence and anergy (84, 85) although it is also regarded as a marker of differentiation in CD8 (86) and NK (87) cells. CD57^+^ NK cells are highly cytotoxic and their presence seems to be beneficial in a number of infectious diseases. However, we observed an inverse correlation between CD57^+^ NK cells and NKp46 expression in the ME/CSF cohort (*p* < 0.0001, rho = −0.58) that is also present in the healthy population (*p* = 0.0075, rho = −0.33) in a lesser extent, although when comparing both correlations with a paired *r* the difference is not statistically significant (*p* < 0.01). We hypothesize that this phenotype of higher CD57^+^ and lower NKp46 expression in NK cells could represent different stages of a chronic viral infection, and together with high NKG2C expression, could be consistent with HCMV infection reactivation or latency, whereas low CD57^+^ and high NKp46 together with low NKG2C expression in NK cells could be associated with reactivation or latency of EBV infection. Our study did not show any differences regarding IgG EBV and HCMV serology between ME/CFS patients and healthy controls. Scheibenbogen et al. (5) had detected EBV DNA sequences in B lymphocytes by EBER DNA technique in ME/CFS patients, that could reveal viral activity although the IgG antibody profile would be similar to healthy controls (88).

We observed a significant correlation between the expression of NKG2C and IgG-HCMV antibodies (HS rho = 0.48, ME/CFS rho = 0.13, *p* = 0.048) so higher values of IgG-HCMVH antibodies would be accompanied with lower NKG2C expression in ME/CFS patients than in the HS cohort.

There were three groups of patients according to the different onset of the symptoms. Some patients referred the initial symptoms soon after an infectious process (*n* = 24); others described the symptoms not related to infections (*n* = 37). A third group (*n* = 15) did not remember how the symptoms started; therefore, we exclude them from this analysis. We compared the cell phenotypes of these two groups vs the healthy individuals and observed significant differences between the groups with regards to CD56^++(high)^ (*p* = 0.1559 and *p* = 0.0036 for the “infection” and “no infection” onset groups, respectively) and NKT-like cells (*p* = 0.0243 and *p* = 0.1041, respectively) (Table 2). One explanation could be due to the exposure to raised levels of catecholamines secondary to the chronic activation of the hypothalamic–pituitary–adrenal axis as described by Loebel et al. (51).

Expression of CD69 in NK cells was higher in the ME/CFS group (*p* = 0.011), corroborating the observations by Curriu et al. (34) and in contrast to the study by Mihaylova et al. (26) in which they found this activation marker to be lower in ME/CFS patients but after mitogen stimulation, as opposed to the non stimulatory conditions of our and Curriu studies. However, Theorell et al. (33) did not observe variations of this marker when analyzing previously frozen PBMC. These contradicting observations could be due to the different methodologies used, to the small size of the cohorts, of patients and/or controls, and to a different profile of ME/CFS cohort. Increased levels have also been found in infectious and autoimmune pathologies (89–92). In a study with rheumatoid arthritis (RA) induced in mice, CD69 functioned as regulator of the autoimmune pathology and inflammation by increasing TGF-beta, a cytokine that has been found to be increased in EM/CFS patients (93–95).

Finally, we observed a descent in T regulatory cells in contrast to other studies, where the T regulatory cells were increased in ME/CFS patients (23) or not different to healthy controls (33). T regulatory cells have been found to be lower in autoimmune conditions, such as systemic lupus erythematosus (96) and active RA (97), as well as inverted Th17/T regulatory cells ratio, with an elevated proinflammatory response (98). In RA, treatment with low dose of methotrexate (MTX) increase levels of T regulatory cells and in immune thrombocytopenic purpura (99) with low doses of rituximab (RTX) together with steroids vs steroids only improve clinical symptoms. In a pilot study with three ME/CFS patients, symptoms improved after treatment with MTX and RTX (100), and in a larger study (101) improvement were seen after treatment with RTX only.

We strongly believe that our results can contribute to the knowledge of the immune pathological processes of the disease and to the diagnosis. In particular, the mathematical model proposed in our study, based on the altered cell subpopulations and their correlations could help facilitate and corroborate, with laboratory studies, the diagnosis of ME/CFS in 3 out of 4 patients that already fulfill the clinical criteria of ME/CFS. Furthermore, the phenotypic characterization of these lymphocyte subpopulations may help to categorize ME/CFS patients that could lead to improve the knowledge and understanding of the pathophysiology of these patients and their treatment. However, more studies are needed to corroborate these findings and to contribute to establish a consensus in diagnosis.

## Ethics Statement

This study was carried out in accordance with the recommendations of Ley General de Sanidad (25/4/1986) Art. 10, with written informed consent from all subjects. The study was approved by the Healthcare Ethics Committee of the Hospital Clinic de Barcelona HCB/2015/0870. All participants gave written informed consent, complying with current legislation.

## Author Contributions

JR, TP, and MG contributed conception and design of the study. JR organized the database. GF performed the statistical analysis. JR wrote the first draft of the manuscript. TP, GF, and MG wrote sections of the manuscript. All authors contributed to manuscript revision, read and approved the submitted version.

## Conflict of Interest Statement

The authors declare that the research was conducted in the absence of any commercial or financial relationships that could be construed as a potential conflict of interest. The reviewer JT and handling Editor declared their shared affiliation.
