# Supplementary material for: Association of T and NK Cell Phenotype With the Diagnosis of Myalgic Encephalomyelitis/Chronic Fatigue Syndrome (ME/CFS)
Source: Front Immunol. 2018 May 9;9:1028. doi: 10.3389/fimmu.2018.01028 (PMC5954087; doi:10.3389/fimmu.2018.01028)
Supplement: Supplementary file 1 [file Data_Sheet_1.docx]

Supplementary Material

**ASSOCIATION OF T AND NK CELL PHENOTYPE WITH THE DIAGNOSIS OF MYALGIC ENCEPHALOMYELITIS/CHRONIC FATIGUE SYNDROME (ME/CFS)**

Jose Luis Rivas* ^1^, Teresa Palencia ^1^, Guerau Fernández ^2^, Milagros García^1,3^

^1^ ME/CFS Unit, ASSSEMBiomédics, Barcelona, Spain

^2^Bioinformatics Unit, Genetics and Molecular Medicine Service, Hospital Sant Joan de Déu, Esplugues del Llobregat, Spain

^3^ Immunology Department, Biomedical Diagnostic Center, Hospital Clínic Barcelona, Spain

# * Correspondence: Jose Luis Rivas binizzy@yahoo.es

# Supplementary Figures and Tables


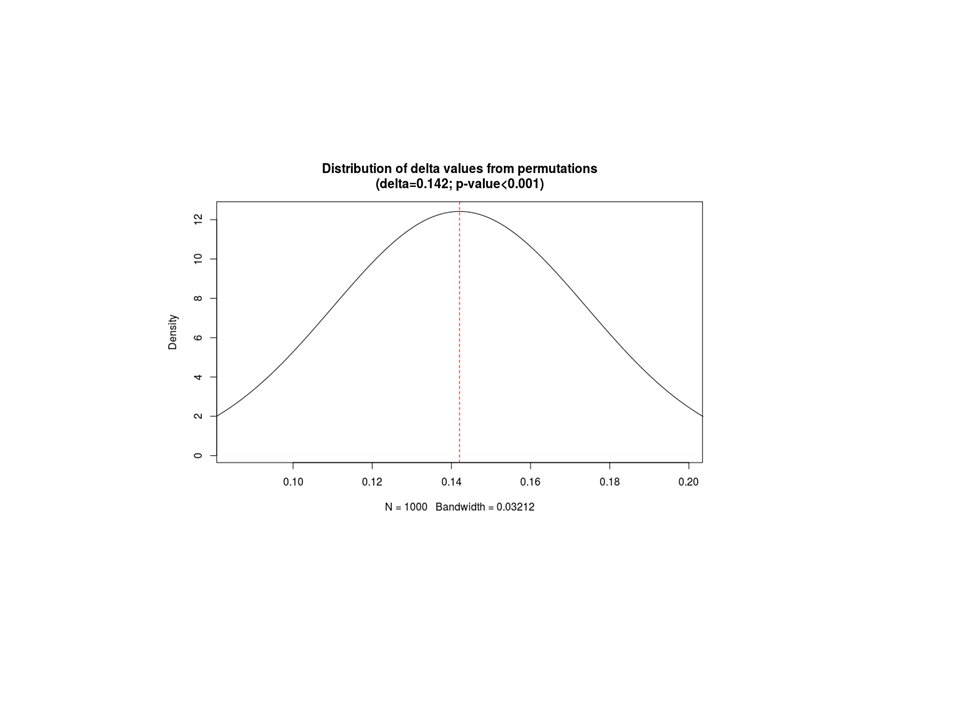


**Supplementary Figure 1**. Delta statistic that quantifies the proportion of variance due to batch effects. Plot of delta values compared to the delta value generated taking into account our extraction grouping (red dashed line). We can observe that our delta value falls within the permutated distribution showing no batch effect due to extraction date.


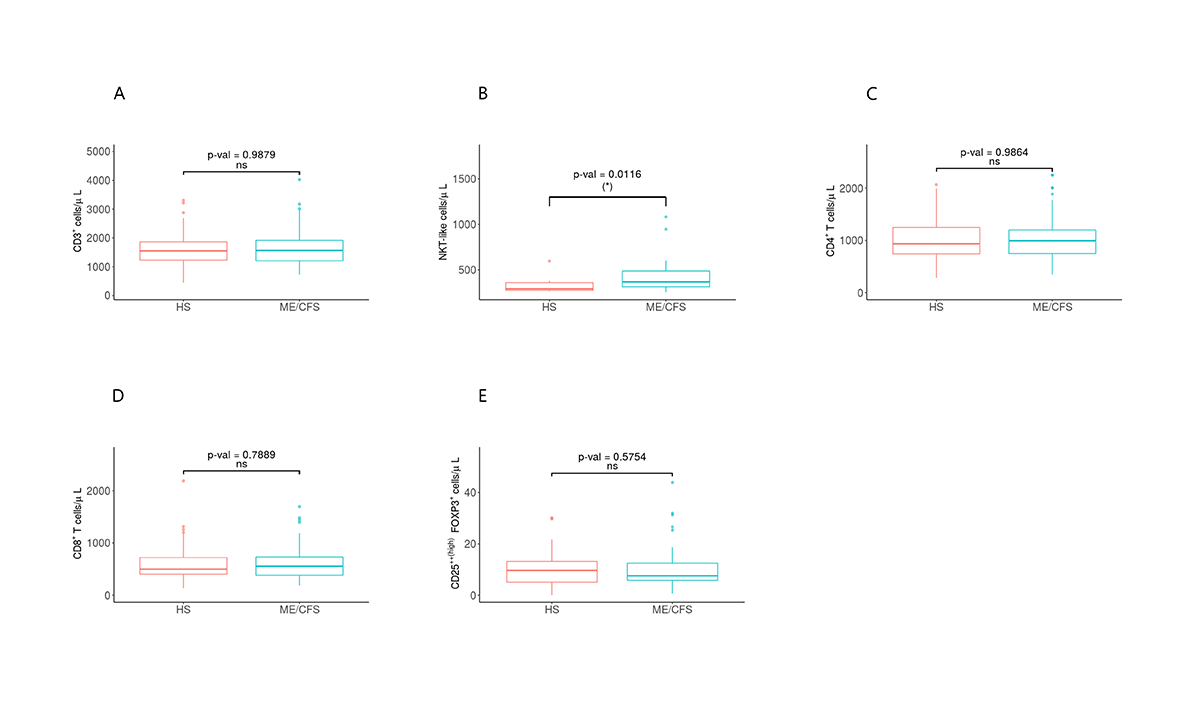


**Supplementary Figure 2.** Comparison of lymphocytes subsets in ME/CFS patients (n=76) and healthy subjects (n=73). **(A)** Absolute count of T (CD3^+^) and **(B)** NKT-like (CD3^+^CD16^±^CD56^+^) cells were calculated from the percentages obtained in gated CD45^+^ lymphocytes and referred to the whole white cell count (WBC). **(C), (D)** Absolute count of CD4^+^ and CD8^+^ cells were calculated from the percentages obtained gating CD3^+^ lymphocytes and referred to the WBC. **(E)** The absolute count of T regulatory cells (CD25^++(high)^FoxP3^+^) was calculated from the percentage obtained gating for CD4^+^ T-cells and referred to the WBC. Figures show median values (lines), interquartile ranges (boxes) and 10–90 percentile values (bars). P values obtained by Wilcoxon non-parametric test.


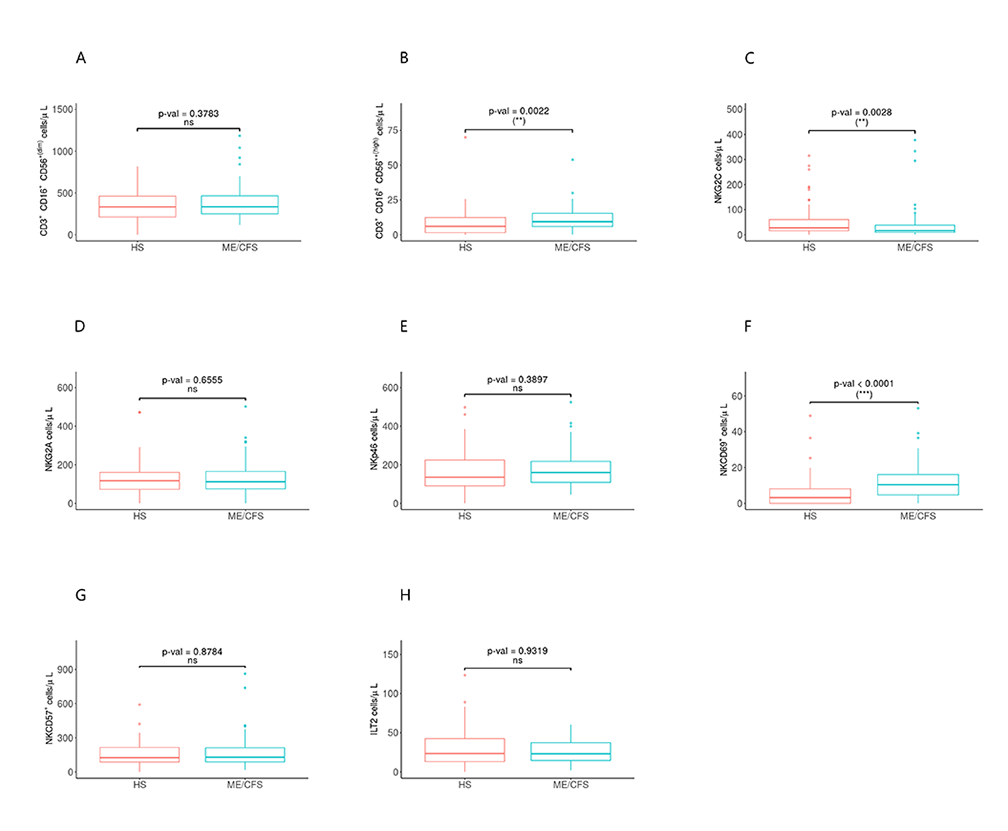


**Supplementary Figure 3.** Analysis of NK cell subsets in ME/CFS patients (n=76) and healthy subjects (n=73). **(A), (B)** Absolute count of NK cells as CD16^±^CD56^+(dim)^ and CD16^±^CD56^++(high)^ were calculated from the percentage obtained in gated CD45^+^ lymphocytes and referred to the total white cell count (WBC). **(C), (D), (E), (F), (G), (H)** Absolute count of NKG2C, NKG2A, NKp46, NKCD69, NKCD57, ILT2 NK cells were calculated from the percentage obtained after gating for CD16^±^CD56^+^ lymphocytes and referred to the WBC. Figures show median values (lines), interquartile ranges (boxes) and 10–90 percentile values (bars). P-values obtained by Wilcoxon non-parametric test.


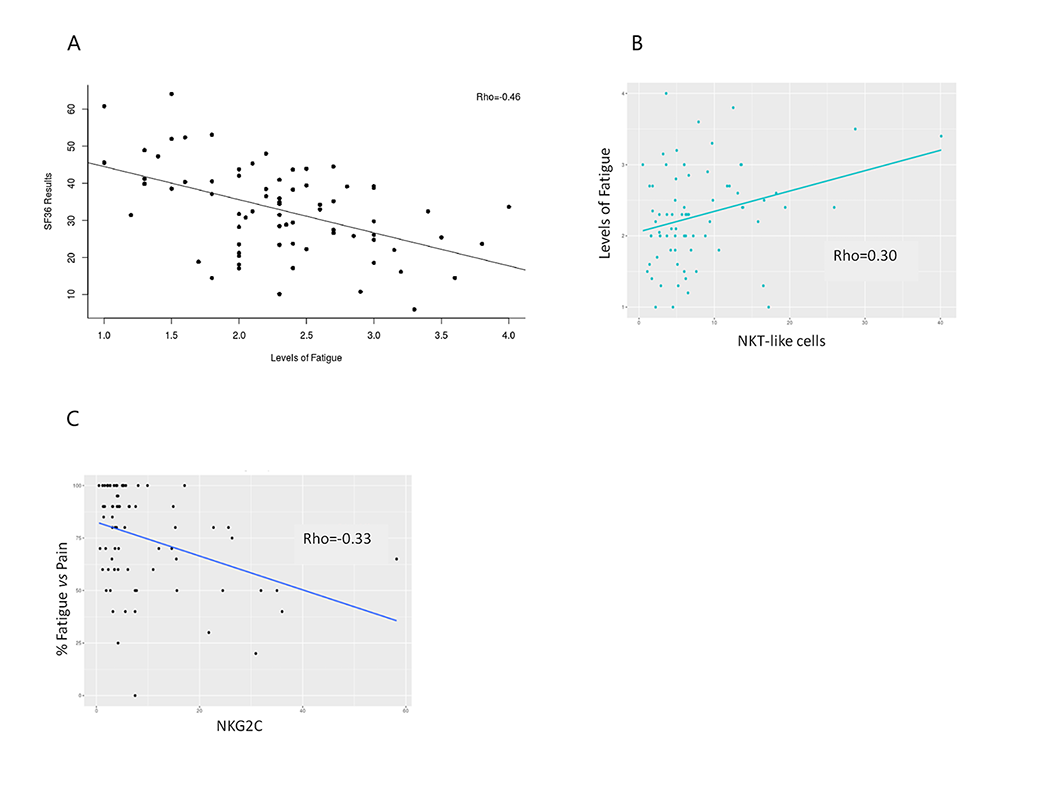


**Supplementary Figure 4.** Correlation analysis. **(A)** Moderate correlation between the levels of fatigue and SF36 questionnaire (Rho=-0.46). Values of SF-36 decrease as the levels of fatigue increase; **(B)** Correlation between levels of fatigue and NKT (CD3^+^CD16^±^CD56^+^) cells. The levels of fatigue rise as the NKT cell population increase; **(C)** Correlation between percentage of fatigue *vs* pain and NKG2C expression. Fatigue is greater than pain when expression of NKG2C is lower.
